# Aspartate-β-Hydroxylase: A Promising Target to Limit the Local Invasiveness of Colorectal Cancer

**DOI:** 10.3390/cancers12040971

**Published:** 2020-04-14

**Authors:** Roberto Benelli, Delfina Costa, Luca Mastracci, Federica Grillo, Mark Jon Olsen, Paola Barboro, Alessandro Poggi, Nicoletta Ferrari

**Affiliations:** 1 SSD Oncologia Molecolare e Angiogenesi, IRCCS Ospedale Policlinico San Martino, largo Rosanna Benzi 10, 16132 Genova, Italy; delfina.costa@hsanmartino.it (D.C.); alessandro.poggi@hsanmartino.it (A.P.); nicoletta.ferrari@hsanmartino.it (N.F.); 2 Anatomia Patologica, IRCCS Ospedale Policlinico San Martino, largo Rosanna Benzi 10, 16132 Genova, Italy; mastracc@hotmail.com (L.M.); federica.grillo@unige.it (F.G.); 3 Anatomia patologica, Dipartimento di Scienze Chirurgiche e Diagnostiche Integrate (DISC), Università di Genova, 16132 Genova, Italy; 4 Department of Pharmaceutical Sciences, Midwestern University, Campus Glendale, Glendale, AZ 85308, USA; molsen@midwestern.edu; 5 Clinica di Oncologia Medica, IRCCS Ospedale Policlinico San Martino, largo Rosanna Benzi 10, 16132 Genova, Italy; paola.barboro@hsanmartino.it

**Keywords:** ASPH, colorectal cancer, cell invasion, tumor budding

## Abstract

Colorectal cancer’s (CRC) ability to invade local tissues and lymph nodes and generate distant metastases is the key for TNM classification. Aspartate-β-hydroxylase (ASPH), a transmembrane protein that catalyzes Notch receptors and ligand activation, is involved in tumor invasion. Because Notch is involved in gut homeostasis, it could be a target for CRC therapy. ASPH mRNA and protein expression, promoter methylation and gene copy numbers were evaluated using the TCGA and CPTAC human CRC datasets. Using digital pathology, ASPH was scored in the luminal area (LM), center tumor (CT) and invasive margin (IM) of 100 human CRCs. The effect of ASPH targeting on invasiveness and viability was tested by siRNA knockdown and small molecule inhibitors (SMI). Bioinformatics analysis showed increased expression of ASPH mRNA and protein in CRC, paired with a decreased methylation profile. ASPH genetic gain or amplification was frequent (56%), while deletion was rare (0.03%). Digital pathology analysis showed that ASPH exerted its pathological activity in the invasive margin of the tumor, affecting invasive front morphology, tumor budding and patients’ overall survival. In vitro, ASPH targeting by siRNA or SMI reduced cell invasion and growth and caused Notch-1 downregulation. This study demonstrates that ASPH targeting by specific inhibitors could improve CRC treatment strategies.

## 1. Introduction

Aspartate-β-hydroxylase (ASPH) is a transmembrane protein that catalyzes the hydroxylation of aspartyl and asparaginyl groups in the epidermal growth factor-like domains of membrane molecules, including Notch and its ligands. Notch, in turn, is involved in the stemness and drug resistance of colorectal cancer cells [1]. ASPH is upregulated in several malignant neoplasms (hepatocellular, pancreatic, lung and neural carcinomas) and correlates with a reduced survival rate [2,3,4,5,6]. In vitro, ASPH overexpression induces a malignant phenotype, characterized by increased motility and matrix invasion [7,8].

A unique study on human tissue microarrays [9] immunohistochemically evaluated the expression of a truncated form of ASPH, Humbug, on a cohort of stage II colorectal carcinoma (CRC). According to this study, Humbug levels would correlate directly with the degree of cell differentiation and inversely with patients’ prognosis, while full-length ASPH would not because its expression is also diffuse in normal mucosa. In this study, the antibody used to detect Humbug recognized the N-terminal domain shared with ASPH; thus, it could not be used to assess specific Humbug involvement. Moreover, Humbug is enzymatically inactive and thus unable to activate the Notch pathway [10]. To obtain a more focused characterization of ASPH involvement in local tissue invasion during primary CRC progression, we coupled a new immunohistochemistry (IHC) evaluation, in which ASPH expression was separately evaluated in the luminal, central and invasive area of the tumor by digital pathology, to in vitro tests. As we show in this brief communication, this approach suggests the active involvement of ASPH at the invasive margin of CRC and thus an opportunity to limit CRC invasion by ASPH silencing or by targeted therapeutic inhibition.

## 2. Results and Discussion

### 2.1. Bioinformatics Analysis Shows ASPH Upregulation in CRC

**Figure 1:**
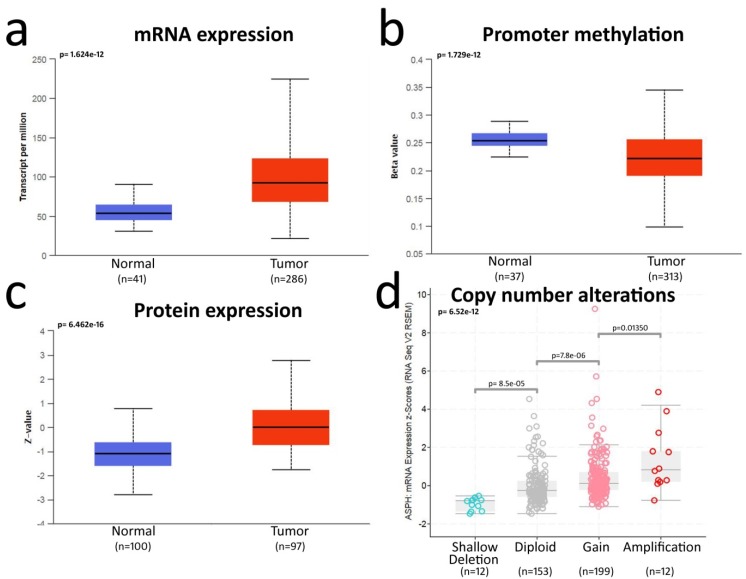
Bioinformatics analysis of aspartate-β-hydroxylase (ASPH) overexpression in CRC: (**a**) analysis of ASPH mRNA expression in normal mucosa and colorectal cancer (CRC) samples from TCGA database (UALCAN website); (**b**) ASPH promoter methylation status in normal mucosa and CRC samples from TCGA database (UALCAN website; beta-value scale ranges from 0 = unmethylated to 1 = fully methylated); (**c**) analysis of ASPH protein levels by mass spectrometry in normal mucosa and CRC samples from the CPTAC database (UALCAN website; Z-values represent standard deviations from the median across the samples); (**d**) putative copy number alterations of the ASPH gene computed by GISTIC algorithm in TCGA database (c-BioPortal website; shallow deletion = 1− copy, gain = 1+ copy, amplification ≥ 2+). Box plots represent the quartile distribution of data around the median value. Statistics are reported in each graph as the calculated *p* value.

Our bioinformatics analysis showed a clear upregulation of ASPH mRNA and protein in CRC as compared to control mucosa (Figure 1a,c). This upregulation is shared by other cancers, such as head and neck squamous cell carcinoma, liver hepatocellular carcinoma, lung adenocarcinoma, lung squamous cell carcinoma and stomach adenocarcinoma (Figure S1).

Accordingly, the ASPH promoter was less methylated in CRC as compared to normal mucosa (Figure 1b). A detailed analysis of the ASPH gene methylation pattern showed that methylation also occurred outside of the ASPH gene promoter and involved single CpG dinucleotides lining regions for alternative splicing. Methylation target sites were superimposable in normal mucosa and CRC (Figure S2). The evaluation of putative ASPH gene copy number alterations by GISTIC showed 56% of CRC samples with gain/amplifications, while shallow deletions were very rare, and complete deletions were not detected (Figure 1d). In CRC, the amplification of the ASPH gene was associated with a cluster of 115 co-amplified genes on chromosome 8 (Figure S3). Interestingly, a small cluster of only eight genes, amplified in the same region in lung cancer, also contained ASPH, suggesting a possible positive selection for ASPH amplification during the progression of these tumors.

These observations do not confirm a previous report describing full-length ASPH as equally expressed in normal mucosa and colorectal cancer [9]. Moreover, the frequent gain of ASPH gene copies and the decreased methylation of the ASPH promoter suggest a positive selection for ASPH upregulation during CRC progression.

**Figure 2:**
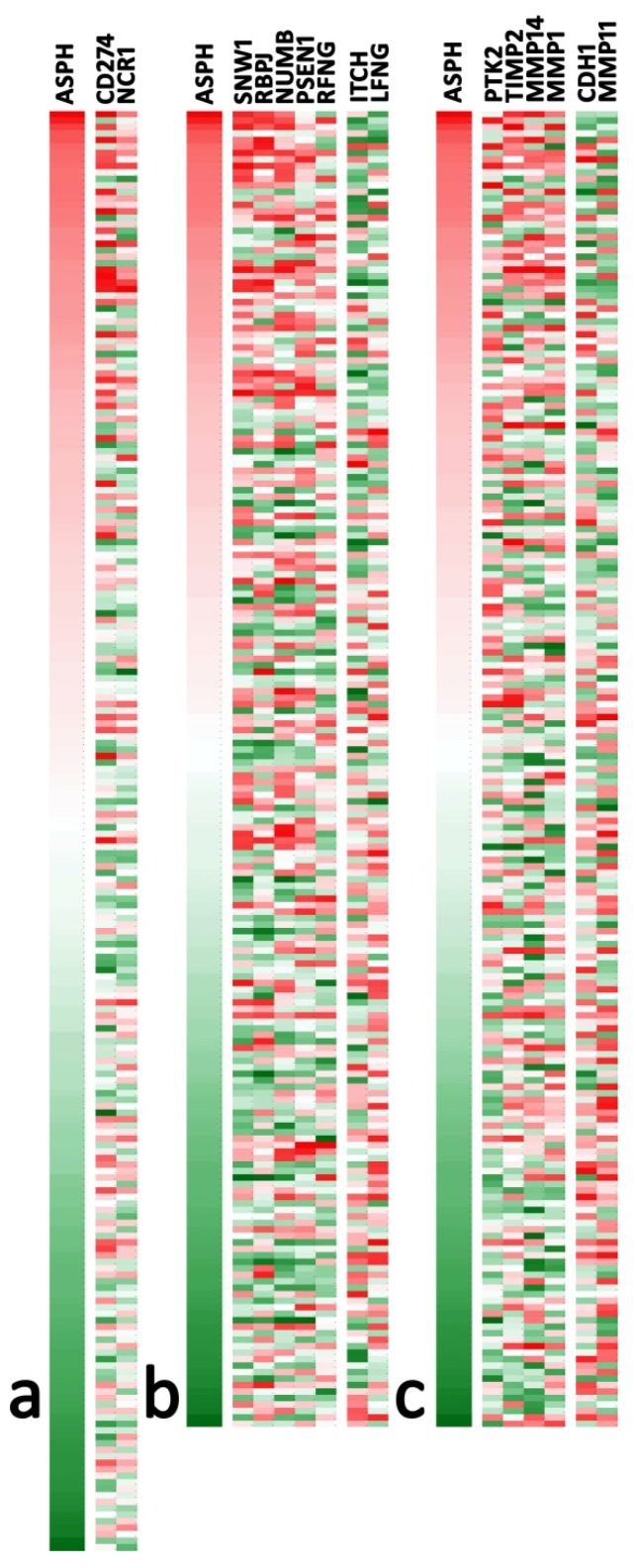
Heatmaps of mRNA-clusters significantly correlated with ASPH expression (red = high, green = low), selected in Table S1: (**a**) Immune signature (*n* = 222); (**b**) Notch signature (*n* = 203); (**c**) Invasive signature (*n* = 203). Each cluster is ordered according to Pearson’s *r* coefficients of each marker against ASPH, from higher (left) to lower (right). The maximum *r* coefficient was 0.330 (SNW1), and the lowest was -0.304 (LFNG); thus, no marker showed a widespread correlation with ASPH among analyzed samples. Microarray *Z*-scores for the selected markers were extracted from the COAD Firehouse legacy database (TCGA) using the cBioPortal web interface.

Using the TCGA COAD database, we also analyzed the relation of ASPH mRNA levels with markers that could influence tumor invasion: the reaction of immune cells, the activation of the Notch pathway and the balance of invasion markers and their inhibitors. Among several markers analyzed (see Table S1), those retaining a statistically significant association with ASPH are reported in Figure 2. Despite relatively low correlation indexes, ASPH levels showed a significant association with several markers, particularly with increased CD274/PDL1 and NCR1/NKp44 in the immune signature and modulation of numerous mRNA associated with the Notch signature. Invasion markers showed upregulated PTK2/Focal adhesion kinase 1, while CDH1/E-Cadherin was downregulated. Matrix metalloproteinases (MMP) 14 and 1 were upregulated along with the MMP2 inhibitor TIMP2, while MMP11 was downregulated. This proteinase/inhibitor balance is difficult to interpret, as both tumor epithelial cells and reactive fibroblasts could be involved.

### 2.2. Digital Pathology Analysis Shows a Specific Role for ASPH in the Invasive Tumor Margin

Given the involvement of ASPH in the modulation of Notch signaling and tissue invasion, as well as its likely relevance in the prognosis and therapeutic targeting of CRC, a more focused pathological evaluation of this marker becomes essential. To this purpose, we selected a monoclonal antibody that specifically recognizes ASPH in its active high molecular weight form (86 KDa). Western blot analyses carried out on CRC cell lines and tissue lysates showed a unique high molecular weight band (Figure S4). We thus used this antibody to histologically evaluate ASPH in 100 CRC samples by scoring ASPH levels separately in the luminal area (LM), center tumor (CT) and invasive margin (IM) (Figure S5) and then assessing its link to invasive pathological parameters, as well as patient prognosis. 

**Figure 3:**
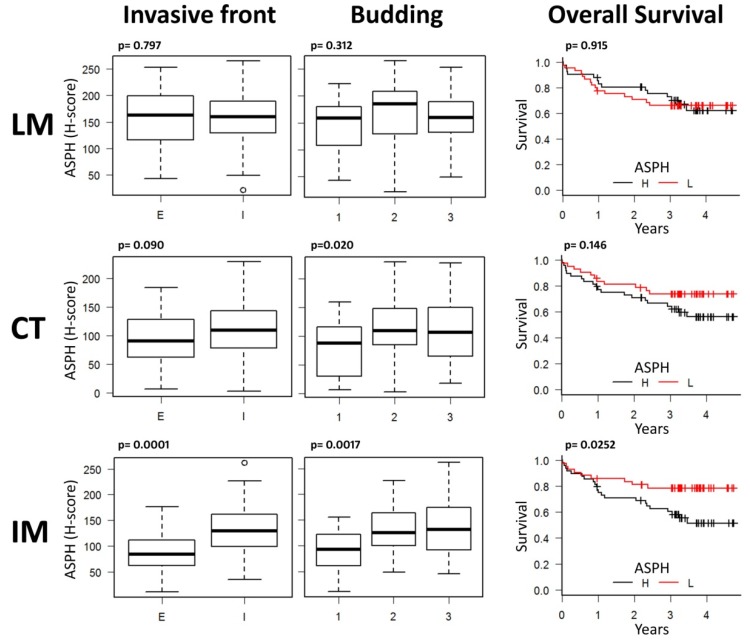
ASPH expression in the tumor invasive margin (IM) correlates with the pattern of local invasion and patients’ overall survival: invasive front type (I = infiltrative, E = expansive), tumor budding (1 = 0–4 buds, 2 = 5–9 buds, 3 ≥ 10 buds) and overall survival (L = low, H = high ASPH levels dichotomized by the median value) show a strong relation only with ASPH levels scored in CRC IM. Center of the tumor (CT)-scored ASPH shows a statistically significant relation only with tumor budding, while ASPH expressed next to tumor lumen (LM) does not show any relation with these parameters. Box plots represent the quartile distribution of ASPH H-score quantifications in CRC samples around the median value. Statistics are reported in each graph as the calculated *p* value (Invasive front: Student *t*-test; Budding: one-way ANOVA; Overall survival: Kaplan–Meier analysis).

CRC samples showed a variable intensity of staining for ASPH, with some samples almost negative and others strongly positive (Figure S6). Normal or hyperplastic mucosa adjacent to the tumor was almost invariably negative or faintly stained (Figure S7), confirming the bioinformatics data. Digital pathology analysis showed that ASPH levels were not uniform in the tumor (Pearson’s correlation coefficient of IM H-scores compared to CT H-scores was 0.63, *p* = 0.000000000018; Pearson’s correlation coefficient of IM H-scores compared to LM H-scores was 0.402, *p* = 0.000124); thus, the quantification of ASPH levels in the whole tumor, used to obtain omics data, does not necessarily reflect the specific content of ASPH in the tumor invasive margin. Indeed, only IM H-scoring gave significant and coherent results (Figure 3). In the IM, ASPH levels were increased in the presence of an infiltrative tumor margin, ≥2–3-scored budding and reduced overall survival (OS). In CT, only the relation with budding retained statistical significance, while ASPH levels did not show any correlation with these parameters in the LM. ASPH IM levels did not correlate with other pathologic parameters (stage *p* = 0.974, grade *p* = 0.479, tumor location *p* = 0.965, perineural invasion *p* = 0.387). The relation of ASPH levels with microsatellite instability (MSI) did not show a statistically significant linkage, though this datum should be confirmed in a dedicated study because of the limited availability of MSI samples, both in our study and in the TCGA-COAD database (Figure S8).

These data suggest that the evaluation of ASPH levels in the invasive margin of CRC and specifically in tumor epithelial cells cannot be compared to a whole-tumor quantification, as ASPH expression is not uniform and could apparently affect only specific pathological parameters of the invasive front. Accordingly, total ASPH mRNA levels evaluated in the TCGA-COAD database did not affect patients’ prognosis (Figure S9).

The link between ASPH upregulation and worse local invasive parameters confirmed that ASPH could be strategically targeted for therapeutic purposes. Thus, we tested different in vitro strategies to limit ASPH activity.

### 2.3. ASPH Downregulation Affects CRC Cell Line Invasion and Growth

**Figure 4:**
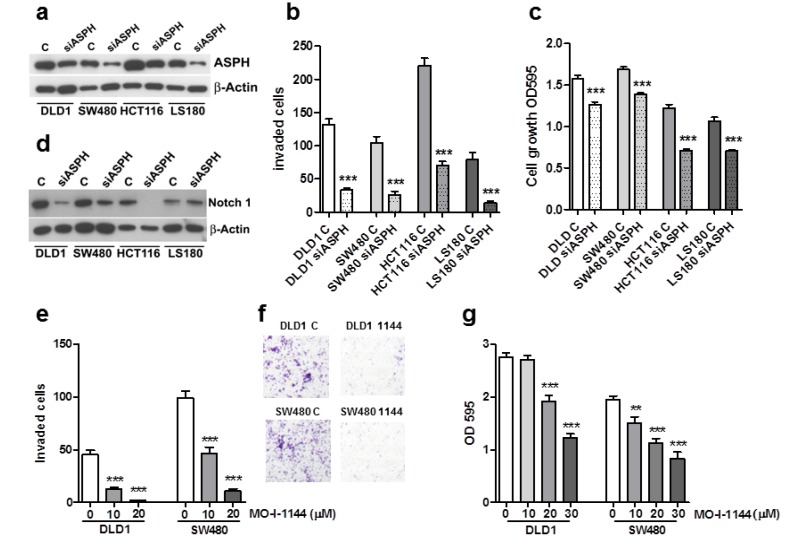
ASPH silencing or activity inhibition decreases cell invasion and proliferation in a panel of CRC cell lines. Specific ASPH siRNAs visibly downregulate ASPH expression after 18 h (**a**). Concomitantly, cell invasion (**b**) and proliferation (**c**) are significantly inhibited. All these effects are associated with Notch1 inhibition (**d**). One representative experiment out of three is reported. β-Actin was utilized as a loading control. Means ± SEM are reported. *** *p* < 0.001. DLD1 and SW480 cell lines were exposed to increasing concentration of MO-I-1144. For the invasion test (**e**), filters were recovered and counted after 18 h. Representative images of filters from 20 µM treated cells are shown in (**f**). Cell growth was evaluated after 96 h by the crystal violet assay (**g**). One representative experiment (conducted on 10 replicates) out of three is reported. Mean ± SEM are reported. ** *p* < 0.01, *** *p* < 0.001.

We reduced ASPH levels by siRNA in a panel of genetically different CRC cell lines (Figure 4a) to confirm its involvement in the invasive phenomenon. Overall, ASPH downregulation strongly inhibited cellular invasion compared to controls (Figure 4b) and also reduced cell proliferation (Figure 4c). ASPH silencing correlated with decreased Notch 1 expression, further confirming that ASPH could affect the Notch pathway in colorectal cancer cells (Figure 4d). Further, ASPH silencing in DLD1 and SW480 cell lines induced a synergistic effect on 5FU (fluorouracil) + Oxaliplatin combined chemotherapy (Table S2). These data show that a partial reduction of ASPH levels could strongly affect CRC invasiveness and also influence cell proliferation. The translation of this therapeutic potential to the patient could be obtained by specific small molecule inhibitors (SMIs).

### 2.4. ASPH Enzymatic Activity Inhibition Decreases CRC Cell Invasion and Growth 

To counteract the ASPH enzymatic activity, six SMIs [6] were tested on the DLD1 and SW480 cell lines, and their effects on cell invasion and growth were quantified. Among these compounds, MO-I-1144 (10–30 µM) caused a dose-dependent reduction of extracellular matrix invasion (Figure 4e,f) and of CRC cell proliferation (Figure 4g). Lower concentrations were ineffective (Figure S10). Of note, both cell lines were more susceptible to invasion inhibition than growth inhibition, in agreement with the pro-invasive property of ASPH and mRNA silencing data. This result supports the need for ASPH enzymatic activity in CRC invasion and the therapeutic potential for targeting.

## 3. Materials and Methods

### 3.1. Bioinformatics Analysis

Data on the differential expression of ASPH mRNA and protein and the ASPH promoter methylation status in normal colorectal mucosa and CRC were obtained by specific queries on the UALCAN web interface (http://ualcan.path.uab.edu), based on TCGA and CPTAC data sets. Putative ASPH gene copy number alterations in CRC were computed by GISTIC using the c-BioPortal web interface (https://www.cbioportal.org) and TCGA data set. Expression data from c-BioPortal were also downloaded to detail the number of samples and perform statistical analysis.

### 3.2. Anti-ASPH Antibody

The anti-ASPH mouse monoclonal antibody (clone A10, IgG1, Santa Cruz, Santa Cruz, CA, USA) was raised against aminoacidic residues 382–681 (which are not shared with the truncated proteins Junctine, Junctate and Humbug, derived from the same gene [10]) and specifically recognized ASPH in its active, high molecular weight form (86 KDa) (Figure S4).

### 3.3. Patient Cohort

This study was approved by the regional ethical committee (Comitato Etico Regionale Liguria) (CER-Liguria 105–2019; 17 June 2019) and applied the European recommendations for data minimization and patient pseudonymization (European Regulation n.679 / 2016 art. 9 and 89; European law 167/2017 art.28). In the Human Protein Atlas database, ASPH has been detected in 100% of CRC samples (2 low, 3 mean, 6 high expression cases of 11 analyzed; https://www.proteinatlas.org/ENSG00000198363-ASPH/pathology); thus, a cohort of 100 CRC samples could reliably represent the variability of this marker (patient list available in Table S3).

Inclusion criteria: 100 formalin-fixed, paraffin-embedded primary CRC samples used for pathological reporting before 2014, containing representative areas of the luminal, central and invasive area of the tumor.

Exclusion criteria: known familiarity (FAP, HNPCC), local recurrence, neuroendocrine tumors, benign adenomas, neo-adjuvant therapy.

Clinical and pathological data were extracted from the pathological report of each case. Tumor budding was re-evaluated in the cohort according to the criteria of the International Tumor Budding Consensus Conference (ITBCC) 2016 [11].

### 3.4. Digital Pathology Analysis

Four-micrometer-thick full sections were cut and mounted on Superfrost slides (Thermo Scientific, Waltham, MA, USA). Immunohistochemistry (IHC) was carried out by an automated Bond-RX immunostainer (Leica, Wetzlar, Germany). An anti-ASPH primary antibody was used at a 1:9000 dilution and detected by the Bond Polymer Refine Detection kit (Leica). Slides were digitalized by an AT2 scanner (Leica) at 20× magnification. The representative regions of interest (ROI, 500 × 500 µm) were identified in the luminal area (LM), central tumor (CT) and invasive margin (IM) of each sample. In the IM, ASPH was quantified in the area with the more advanced front of tumor invasion. Genie and Cytoplasmic v2 macros of Image-scope software (Leica) were trained and combined to obtain the quantification of ASPH expressed only by tumor epithelial cells. The Genie training set contained 74 areas from 5 CRCs with different epithelial and stromal morphologies and showed a specificity of 93% and sensitivity of 84.7% for tumor cells. All slides were revised at the end of the automatic analysis, and manual annotation was applied for the few samples showing a suboptimal identification of tumor cells. Despite this correction, the scoring of ASPH in the same sample was not modified substantially, confirming the good performance and a compensatory effect of our combined macro system. The percentage of ASPH-positive cells and the intensity of staining were reported as H-score (=3x %high + 2x %middle + 1x %low intensity stained cells; 0–300 range), which was used for the comparison with pathological data. For the evaluation of OS, ASPH levels were dichotomized by the median of data.

### 3.5. Cell Lines

DLD1, SW480, HCT116 and LS180 CRC cell lines were obtained from the Biological Bank of our institute (http://www.iclc.it) and cultured in 10% FCS-containing RPMI medium.

### 3.6. ASPH Silencing, Cell Proliferation and Invasion Assays

On-target plus ASPH smart pool siRNA were obtained from Dharmacon (Lafayette, CO, USA) and utilized as suggested by the manufacturer. In vitro cell proliferation analysis was performed in 96-well plates with 500 cells/well, grown in regular medium or treated as described. The number of viable cells was evaluated by the crystal violet assay after 96 h. Cell invasion assay was carried out in cell-Matrigel chambers (BD Bio Coat, Bedford, MA, USA) with 50,000 cells/well following the manufacturer’s protocol. A colon fibroblast-conditioned medium was utilized as a chemoattractant. The test lasted 18 h, and under this experimental condition, the crystal violet assay showed 100% cell viability.

### 3.7. ASPH Inhibitors

A panel of six SMIs of ASPH enzymatic activity, kindly provided by Dr. Mark Olsen (chemical structures are shown in Figure S11), was preliminarily tested to study the effects of ASPH inhibition on cell viability and invasiveness. The first-generation compound MO-I-1100, second generation compounds MO-I-1144, MO-I-1150, MO-I-1151 [5,6,12] and third generation compounds MO-I-1182 and MO-I-1187 were tested. MO-I-1144 proved to be the only one effective in CRC models (Figure S10).

### 3.8. Protein Extraction and Western Blot

Proteins were obtained from control and silenced cells by lysis in protease inhibitors containing RIPA buffer and quantified with the DC Protein Assay kit (Bio-Rad, Hercules, CA, USA). Cell lysates (4 µg/lane) were separated by SDS-PAGE, transferred to PVDF and probed at 4 °C overnight with the specific anti-ASPH antibody (Santa Cruz) or the anti Notch1 antibody (clone E1F11, Cell Signaling Technologies, Danvers, MA, USA). Protein bands were detected by chemiluminescent HRP substrate (Immobilon Western, Millipore, Burlington, MA, USA) and Hyper film-ECL (GE-healthcare, Chicago, IL, USA).

### 3.9. Statistical Analysis

The EZR free software was used for statistical analysis (http://www.jichi.ac.jp/saitama-sct/SaitamaHP.files/statmed.html). The analysis of two groups of data was performed by Student’s *t*-test. The analysis of variance among three or more groups of data was performed by a one-way ANOVA test or Kruskal–Wallis test. Correlations were calculated by Pearson’s or Spearman’s test. Patients’ OS was analyzed by Kaplan–Meier and log-rank test. A *p* ≤ 0.05 was considered statistically significant.

## 4. Conclusions

ASPH is a highly conserved β-dioxygenase enzyme that is overexpressed in several cancer types [13]. ASPH plays important roles in tumor progression through the activation of the Notch signaling pathway, disruption of mitochondrial functions and inhibition of the antitumor activity of natural killer lymphocytes [4]. These pro-tumor activities could mediate the unfavorable prognostic effect of ASPH overexpression in renal, cervical, pancreatic, lung and thyroid cancer (protein atlas database: https://www.proteinatlas.org/ENSG00000198363-ASPH). Little data is available concerning the involvement of ASPH expression in CRC. Wang and colleagues reported no relationship between ASPH and tumor grade or patients’ survival, with the truncated, catalytically inactive form Humbug serving as a potential prognostic biomarker of stage II CRC [9]. Yet, the specific role of ASPH overexpression in CRC invasion and its possible targeting have never been reported. Our data show that ASPH mRNA and protein are overexpressed in CRC compared to normal mucosa; this gain is frequently mediated by increased gene copy numbers, and it also involves promoter demethylation. The rarity of ASPH shallow gene deletions and the absence of complete deletions sustain the pathologic role of ASPH in CRC development. Using an antibody targeting an ASPH domain that is not present in truncated forms, we show that only the fraction of ASPH expressed in the invasive margin correlated with other invasive parameters and affected patients’ OS. In vitro, both ASPH silencing and inhibition strongly affected the invasive ability of CRC cell lines, possibly through Notch modulation.

From these data, ASPH appears to be a promising therapeutic target expressed at low levels in normal colon cells, as well as in most normal adult cells and tissues, except placental trophoblastic cells [14]. As ASPH is a trans-membrane protein expressed on the endoplasmic reticulum and on the surface of malignant cells, the catalytic activity could be targeted by small molecular inhibitors or immunotherapy. Recent advances in the knowledge of ASPH active site conformation and substrate binding [15] provide useful tools for the development of a new generation of targeted inhibitors.

## Supplementary Materials

The following are available online at https://www.mdpi.com/2072-6694/12/4/971/s1: Figure S1: ASPH mRNA levels in normal and tumor tissues; Figure S2: Methylation status of ASPH gene in CRC and normal colon mucosa; Figure S3: Chromosomal regions identifying clusters of genes potentially co-amplified with ASPH in human cancers; Figure S4: ASPH antibody characterization; Figure S5: Example of digital pathology analysis of the luminal area (LM), central tumor (CT) and invasive margin (IM) of a CRC specimen, stained by anti-ASPH antibody; Figure S6: Examples of CRC samples with null/low (left) or high (right) ASPH expression; Figure S7: Normal and hyperplastic mucosa is negative or faintly stained for ASPH, as compared to the adjacent CRC; Figure S8: Analysis of ASPH levels in CRC samples with microsatellite instability (MSI); Figure S9: Kaplan–Meier analysis of overall survival in TCGA-COAD patients; Figure S10: Screening of SMIs on ASPH catalytic activity in CRC cell lines SW480 and DLD1; Figure S11: Structures of ASPH SMIs, Table S1: Evaluation of Immune, NOTCH and Invasive mRNA signatures, according to ASPH levels, in COAD (Firehouse legacy) TCGA samples, Table S2: ASPH silencing in combination therapy, Table S3: Patients list.
