# Erratum: Benelli, R., et al. Aspartate-β-Hydroxylase: A Promising Target to Limit the Local Invasiveness of Colorectal Cancer. *Cancers* **2020**, *12*, 971

**DOI:** 10.3390/cancers12051226

**Published:** 2020-05-13

**Authors:** Roberto Benelli, Delfina Costa, Luca Mastracci, Federica Grillo, Mark Jon Olsen, Paola Barboro, Alessandro Poggi, Nicoletta Ferrari

**Affiliations:** 1 SSD Oncologia Molecolare e Angiogenesi, IRCCS Ospedale Policlinico San Martino, largo Rosanna Benzi 10, 16132 Genova, Italy; delfina.costa@hsanmartino.it (D.C.); alessandro.poggi@hsanmartino.it (A.P.); nicoletta.ferrari@hsanmartino.it (N.F.); 2 Anatomia Patologica, IRCCS Ospedale Policlinico San Martino, largo Rosanna Benzi 10, 16132 Genova, Italy; mastracc@hotmail.com (L.M.); federica.grillo@unige.it (F.G.); 3 Anatomia patologica, Dipartimento di Scienze Chirurgiche e Diagnostiche Integrate (DISC), Università di Genova, 16132 Genova, Italy; 4 Department of Pharmaceutical Sciences, Midwestern University, Campus Glendale, Glendale, AZ 85308, USA; molsen@midwestern.edu; 5 Clinica di Oncologia Medica, IRCCS Ospedale Policlinico San Martino, largo Rosanna Benzi 10, 16132 Genova, Italy; paola.barboro@hsanmartino.it

The authors wish to make the following corrections to this paper [1]:

Please note that Midwestern University signed the technology transfer agreement very recently, this is the reason why we forgot to modify the conflict of interest statement in the last revision of the manuscript.

The “Conflicts of Interest” statement should be changed to:

**Conflicts of Interest:** M.J.O. is the CEO of Crenae Therapeutics which has licensed ASPH inhibitor technologies from Midwestern University. The other authors declare no conflict of interest.

The authors would like to apologize for any inconvenience caused to the readers by these changes.
