# Supplementary material for: Aspartate-β-Hydroxylase: A Promising Target to Limit the Local Invasiveness of Colorectal Cancer
Source: Cancers (Basel). 2020 Apr 14;12(4):971. doi: 10.3390/cancers12040971 (PMC7226058; doi:10.3390/cancers12040971)
Supplement: Supplementary file 1 [file cancers-12-00971-s001.pdf]

Supplementary Materials

Aspartate-β-Hydroxylase: A Promising Target to Limit the Local Invasiveness of Colorectal Cancer

Roberto Benelli, Delfina Costa, Luca Mastracci, Federica Grillo, Mark Jon Olsen, Paola Barboro, Alessandro Poggi and Nicoletta Ferrari

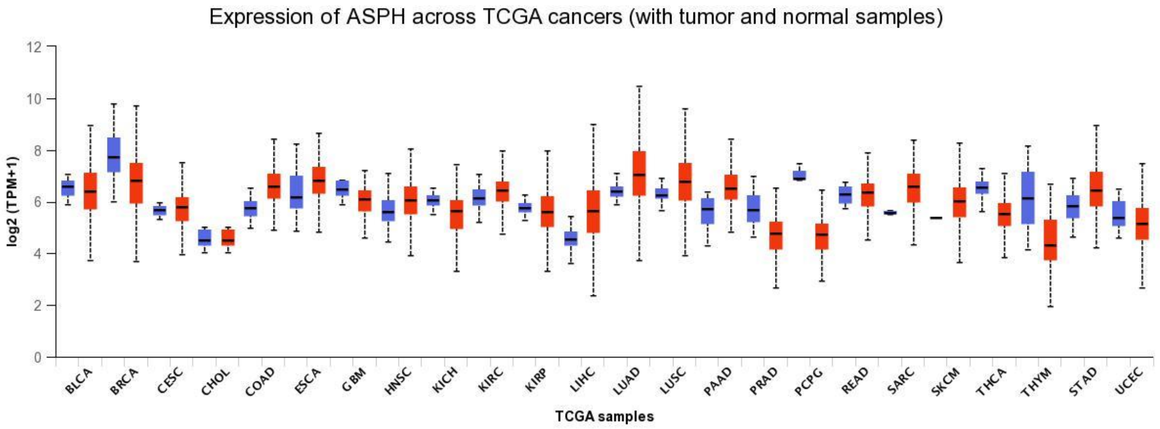

Figure S1. ASPH mRNA levels in the normal tissue (blue) and the related cancer (red) of all available TCGA samples (analysis by UALCAN website). Samples with a highly statistically significant increase of ASPH mRNA levels in tumor tissue ($p < 0.0001$) are highlighted in bold in the list below. The number of normal and tumor samples available for comparison is indicated (vs = versus).

BLCA	bladder urothelial carcinoma,	19 vs 408
BRCA	breast invasive carcinoma,	114 vs 1097
CESC	cervical squamous cell carcinoma,	3 vs 305
CHOL	cholangiocarcinoma,	9 vs 36
**COAD**	**colon adenocarcinoma,**	**41 vs 286**
ESCA	esophageal carcinoma,	11 vs 184
GBM	glioblastoma multiforme,	5 vs 156
**HNSC**	**head and neck squamous cell carcinoma,**	**44 vs 520**
KICH	kidney chromofobe,	25 vs 67
KIRC	kidney clear cell carcinoma,	72 vs 533
KIRP	kidney papillary cell carcinoma,	32 vs 290
**LIHC**	**liver hepatocellular carcinoma,**	**50 vs 371**
**LUAD**	**lung adenocarcinoma,**	**59 vs 515**
**LUSC**	**lung squamous cell carcinoma,**	**52 vs 503**
PAAD	pancreatic adenocarcinoma,	4 vs 178
PRAD	prostate adenocarcinoma,	52 vs 497
PCPG	pheochromocytoma and paraganglioma,	3 vs 179
READ	rectal adenocarcinoma,	10 vs 166
SARC	sarcoma,	2 vs 260
SKCM	skin cutaneous melanoma,	1 vs 472
THCA	thyroid carcinoma,	59 vs 505
THYM	thymoma,	2 vs 120
**STAD**	**stomach adenocarcinoma,**	**34 vs 415**
UCEC	uterine corpus endometrial carcinoma,	35 vs 546

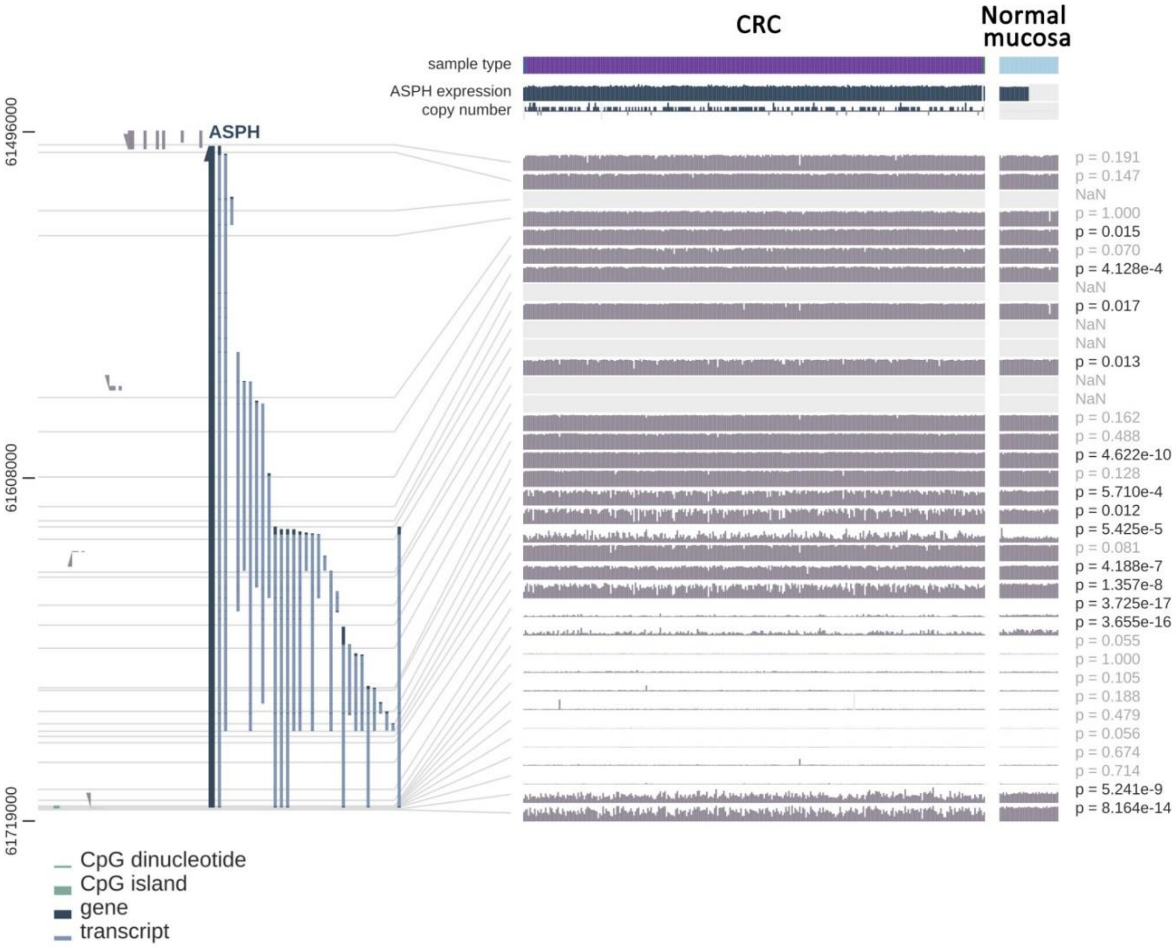

Figure S2. Comparison of the methylation status of each region of ASPH gene in CRC and normal colon mucosa. The analysis shows that ASPH gene promoter is not the only target of a differential methylation. Methylation involves also single CpG dinucleotides probably regulating alternative splicing. CRC and normal mucosa share the same sites of methylation (*p* reported on the right for each putative site of methylation). Analysis performed by MEXPRESS (https://mexpress.be).

ASPH (chr8:62413114-62627199)

Cancer Subset	In Peak?	Nearest Peak (click link to launch IGV)	#Genes in Peak	Q-value	Frequency of Amplification		
					Overall	Focal	High-level
all_cancers	No	chr8:55071319-55382067	1	7.54E-5	0.3692	0.0402	0.0715
Breast invasive adenocarcinoma	Yes	chr8:58428403-63980308	19	1.53E-4	0.5231	0.0778	0.1611
Lung adenocarcinoma	Yes	chr8:61241324-63232864	8	0.00301	0.4845	0.0678	0.0698
Colorectal cancers	Yes	chr8:42886483-71491765	115	0.0196	0.5398	0.039	0.1285
Colon adenocarcinoma	Yes	chr8:52559122-76701065	130	0.158	0.5289	0.0356	0.1222
Rectum adenocarcinoma	Yes	chr8:41702356-96047933	249	0.321	0.5697	0.0485	0.1455
Testicular germ cell tumors	Yes	chr8:54816621-99935217	233	0.52	0.8133	0.0467	0.1
Melanomas	Yes	chr8:42933372-95556884	226	0.957	0.4955	0.0291	0.1435
Sarcoma	Yes	chr8:42933372-145232496	548	1.0	0.3164	0.0703	0.043
Cutaneous melanoma	Yes	chr8:42701314-95556884	230	1.0	0.4672	0.0328	0.0956
Epithelial cancers	No	chr8:55091227-55352528	0	3.8E-6	0.4153	0.0455	0.0834
Ovarian serous cystadenocarcinoma	No	chr8:55186953-55361490	0	9.14E-4	0.5095	0.1589	0.1382
Uveal melanoma	No	chr8:66582149-95556550	147	0.0111	0.625	0.0125	0.3625
Lung cancers	No	chr8:80276890-81835247	7	0.041	0.5034	0.0659	0.056
Kidney chromophobe	No	No peak on chromosome	0	0.797	0.2727	0.0	0.0758
Thymoma	No	No peak on chromosome	0	1.0	0.0813	0.0	0.0081
Adrenocortical carcinoma	No	No peak on chromosome	0	1.0	0.4111	0.0111	0.1889
Mesothelioma	No	No peak on chromosome	0	1.0	0.1609	0.0	0.0
Thyroid carcinoma	No	No peak on chromosome	0	1.0	0.014	0.0	0.0
Liver hepatocellular carcinoma	No	chr8:66441193-95556588	149	1.0	0.4973	0.027	0.1946
Pancreatic adenocarcinoma	No	chr8:40429030-40765717	1	1.0	0.2609	0.0163	0.0054
Pheochromocytoma and Paraganglioma	No	No peak on chromosome	0	1.0	0.0802	0.0062	0.0
Blood cancers	No	chr8:112651697-145232496	233	1.0	0.1255	0.0042	0.0042
Kidney cancers	No	chr8:95338711-145232496	325	1.0	0.1179	0.0	0.0102
Glial cancers	No	chr8:127927969-131798497	22	1.0	0.0936	0.0018	0.0037
Bladder urothelial carcinoma	No	chr8:41492054-42099360	7	1.0	0.5343	0.0711	0.0735
Cervical squamous cell carcinoma	No	chr8:40913581-42701314	20	1.0	0.3085	0.0271	0.0373
Cholangiocarcinoma	No	No peak on chromosome	0	1.0	0.2778	0.0	0.0278
Diffuse large B-cell lymphoma	No	chr8:126156754-145232496	173	1.0	0.125	0.0	0.0
Esophageal carcinoma	No	chr8:80668048-81978492	7	1.0	0.5761	0.0489	0.0761
Glioblastoma multiforme	No	chr8:127927969-131798497	22	1.0	0.0919	0.0	0.0035
Head and neck squamous cell carcinoma	No	chr8:49270178-49935303	4	1.0	0.6054	0.0192	0.067
Kidney renal clear cell carcinoma	No	chr8:111877123-145232496	233	1.0	0.1212	0.0	0.0038
Kidney renal papillary cell carcinoma	No	No peak on chromosome	0	1.0	0.0764	0.0	0.0069
Acute myeloid leukemia	No	No peak on chromosome	0	1.0	0.1257	0.0052	0.0052
Brain lower grade glioma	No	chr8:113046041-138577525	106	1.0	0.0955	0.0039	0.0039
Lung squamous cell carcinoma	No	chr8:42284394-42424881	2	1.0	0.523	0.0639	0.0419
Prostate adenocarcinoma	No	chr8:48689366-61613025	52	1.0	0.2581	0.0264	0.0447
Stomach adenocarcinoma	No	chr8:76288959-77659134	4	1.0	0.5646	0.0499	0.0612
Uterine corpus endometrioid carcinoma	No	chr8:55149374-55384213	1	1.0	0.321	0.026	0.0612
Uterine carcinosarcoma	No	chr8:54410905-56627452	9	1.0	0.6607	0.1429	0.3036

Figure S3. Analysis of chromosomal regions identifying clusters of genes potentially co-amplified with ASPH in human cancers. Analysis performed by the Broad Institute web platform (http://portals.broadinstitute.org/tcga). Please find below the list of the 115 amplified genes clustering with ASPH in the peak region, in CRC. In the red square is highlighted the small cluster of 8 genes co-amplified in lung cancer.

FNTA	CHCHD7	PDE7A
POMK	SDR16C5	DNAJC5B
HGSNAT	SDR16C6P	TRIM55
POTEA	PENK	CRH
LINC00293	LINC00968	LINC00967
RP11-1134I14.8	IMPAD1	RP11-346I3.4
SPIDR	RP11-513O17.2	RRS1
CEBPD	RP11-513O17.3	ADHFE1
PRKDC	LINC00588	C8ORF46
MCM4	FAM110B	MYBL1
UBE2V2	LOC101929528	VCPIP1
RP11-770E5.1	UBXN2B	C8ORF44
LOC101929217	CYP7A1	C8ORF44-SGK3
EFCAB1	SDCBP	SGK3
SNAI2	NSMAF	PTTG3P
C8ORF22	TOX	MCMDC2
SNTG1	CA8	SNHG6
PXDNL	RP11-163N6.2	SNORD87
PCMTD1	RAB2A	TCF24
ST18	CHD7	PPP1R42
FAM150A	LOC100130298	COPS5
RB1CC1	CLVS1	CSPP1
NPBWR1	ASPH ✓	ARFGEF1
OPRK1	MIR4470	CPA6
ATP6V1H	NKAIN3	PREX2
RGS20	UG0898H09	LOC286189
TCEA1	GGH	C8ORF34
LYPLA1	TTPA	RP11-600K15.1
MRPL15	YTHDF3-AS1	RP11-744J10.3
SOX17	YTHDF3	SULF1
RP1	RP11-579E24.1	SLCO5A1
XKR4	LINC00966	PRDM14
SBF1P1	MIR124-2	NCOA2
TMEM68	LOC401463	RP11-333A23.4
TGS1	BHLHE22	TRAM1
LYN	CYP7B1	
RPS20	LINC00251	
SNORD54	LOC286186	
MOS	ARMC1	
PLAG1	MTFR1	

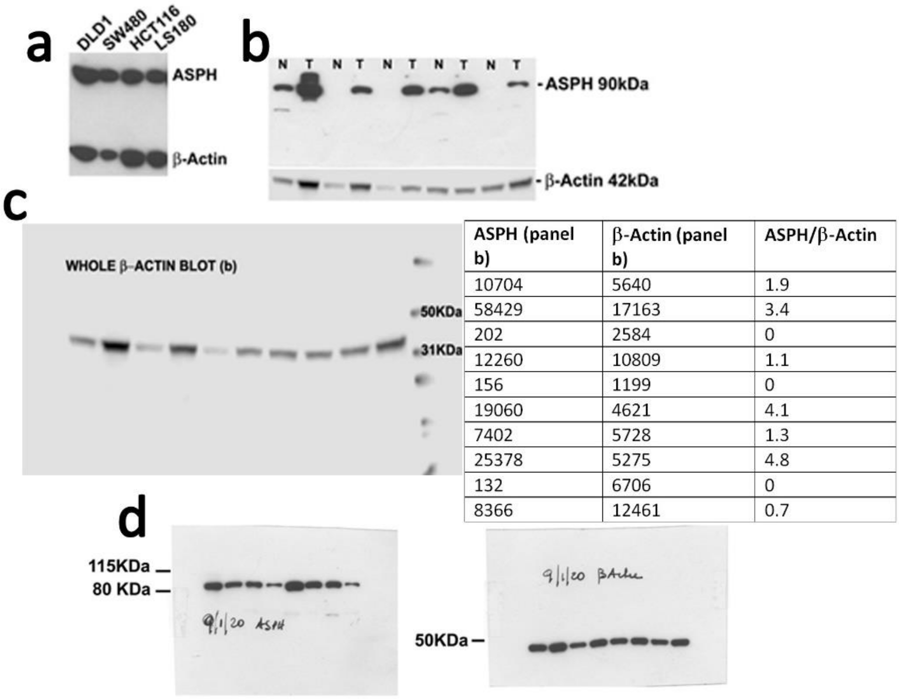

ASPH (panel b)	β-Actin (panel b)	ASPH/β-Actin
10704	5640	1.9
58429	17163	3.4
202	2584	0
12260	10809	1.1
156	1199	0
19060	4621	4.1
7402	5728	1.3
25378	5275	4.8
132	6706	0
8366	12461	0.7

ASPH	β-Actin	ASPH/βActin
1 51610.844	1 48669.945	1 1.1
2 36910.853	2 60720.995	2 0.6
3 36153.581	3 32492.388	3 1.1
4 9690.146	4 46871.167	4 0.2
5 62207.945	5 38468.388	5 1.6
6 43326.095	6 44268.388	6 1
7 38885.752	7 34269.681	7 1.1
8 15166.024	8 42042.095	8 0.4

Figure S4. ASPH antibody characterization: (**a**) Western blot analysis of ASPH expression in lysates from CRC cell lines (whole blot for both ASPH and beta-actin proteins is shown); (**b**)Western blot analysis of ASPH expression in tissue samples (whole blot is shown for ASPH protein). N, normal tissue; T, tumor tissue. Long exposures reveal a unique band corresponding to the active high molecular weight form (86kDa) of ASPH. (**c**) Whole blot of beta actin and table with desitometric values of ASPH, and their ratio with β-actin, reported in order of appearance; (**d**) Full size images of blots shown in figure 4 and their densitometric quantification. Quantifications are shown in order of appearance.

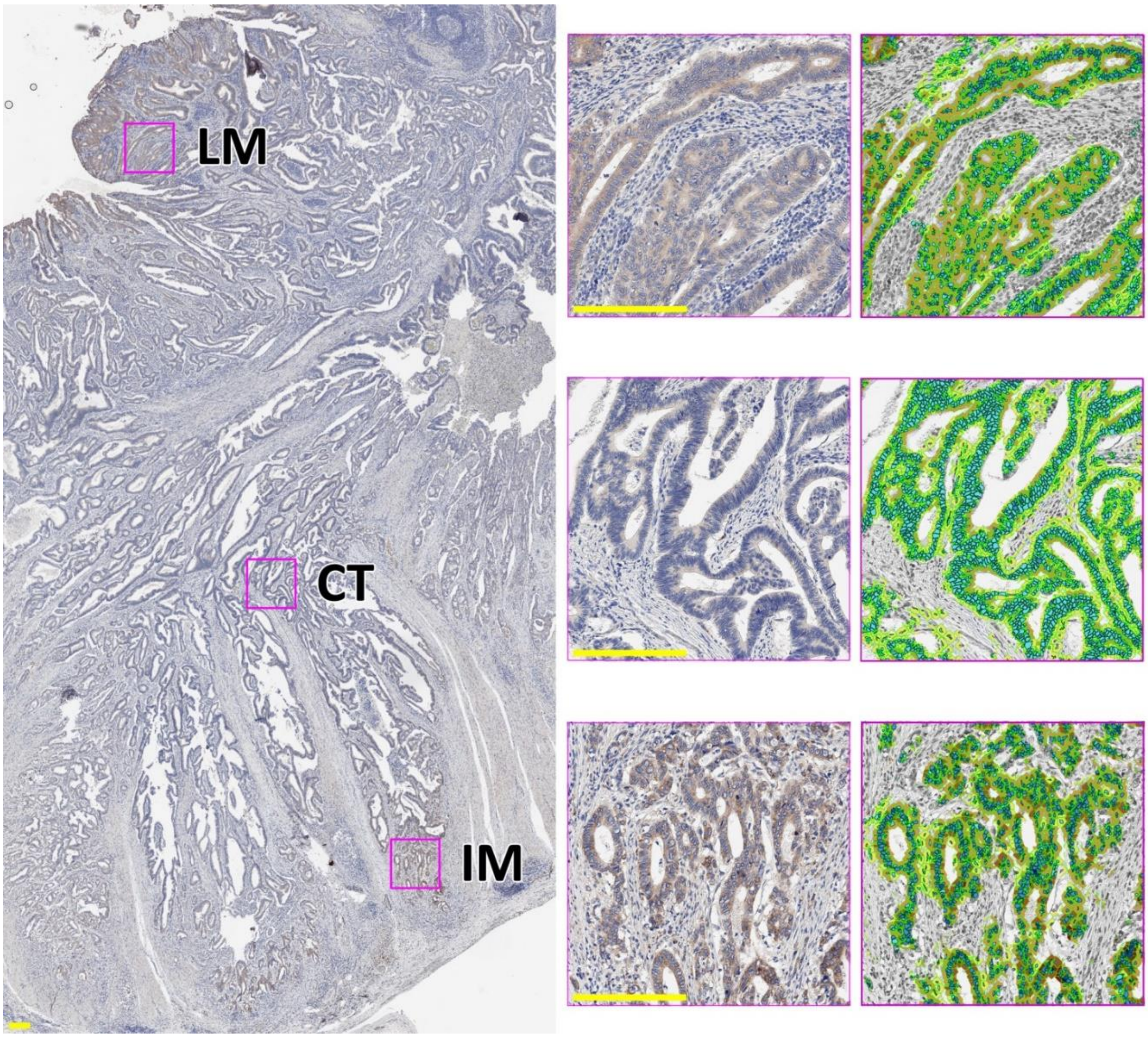

Figure S5. Example of digital pathology analysis of the luminal area (LM), central tumor (CT) and invasive margin (IM) of a CRC specimen, stained by anti ASPH antibody. Annotated regions of interest (ROI) are shown in pink squares. The recognition of tumor cells and the resultant H-score quantification of ASPH was obtained combining a trained Genie algorithm with a modified Cytoplasmic v2 macro of the Image Scope software. Scale bar: 200 μm.

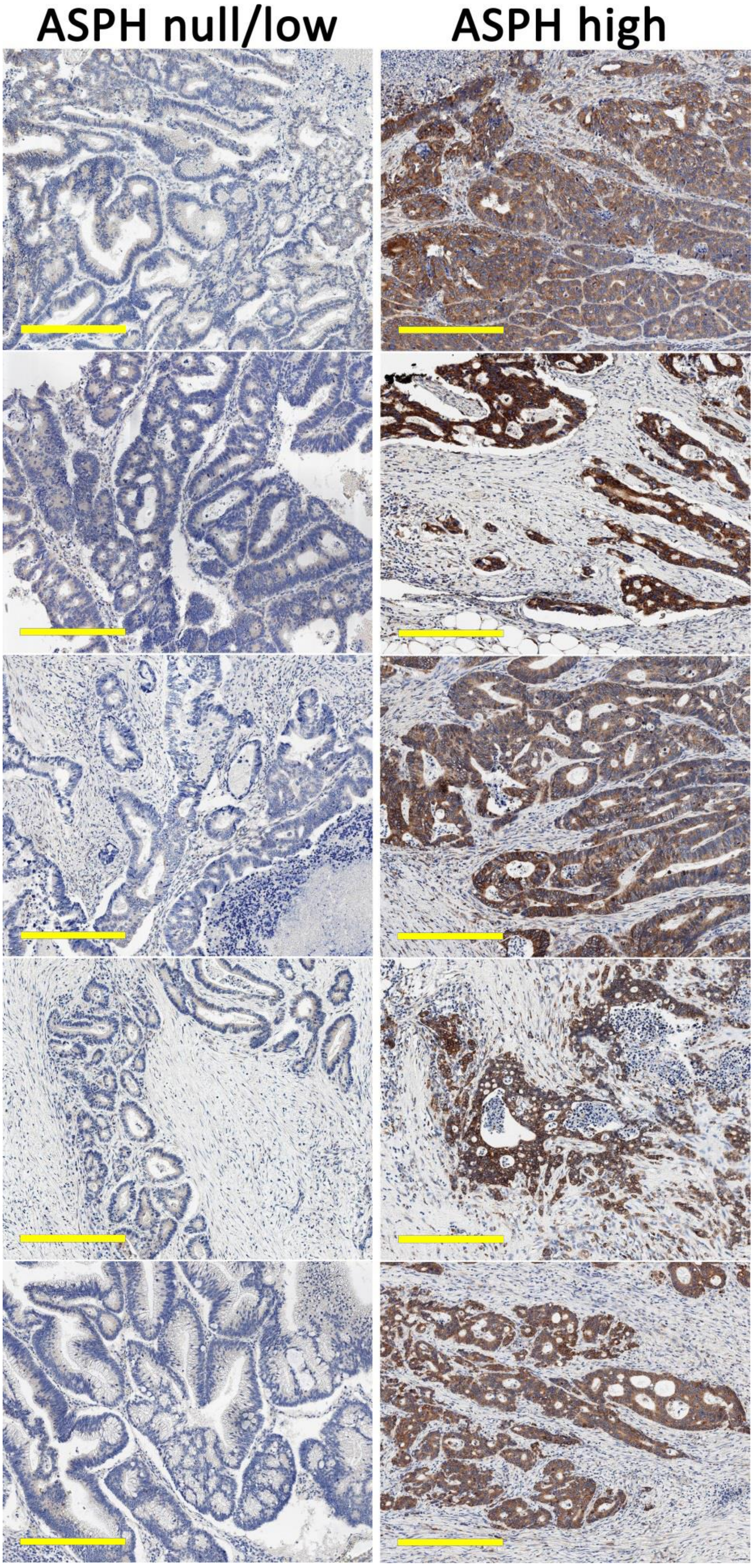

Figure S6. Examples of CRC samples with null/low (left), or high (right) ASPH expression. Scale bar: 300 μm.

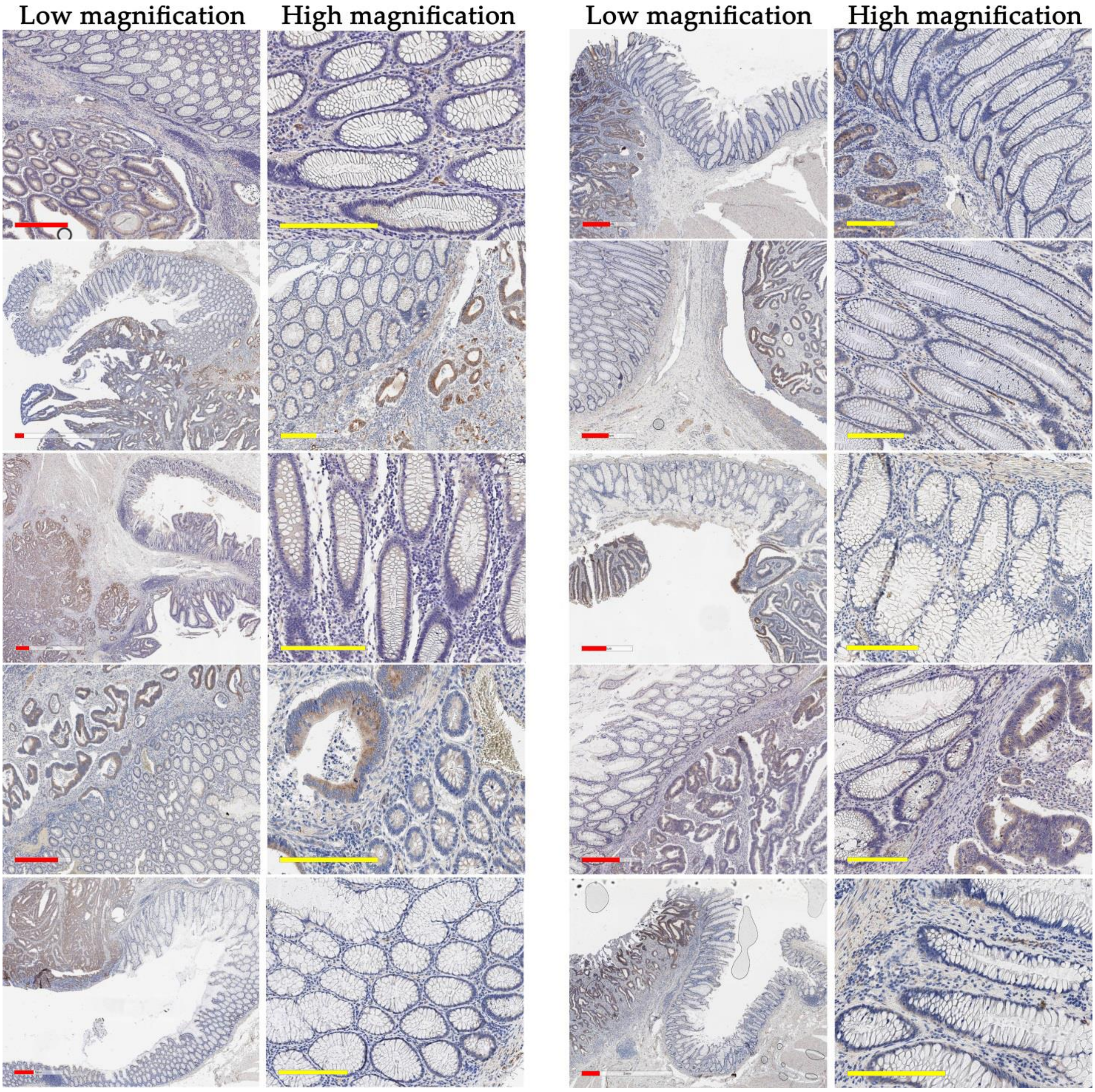

Figure S7. Normal and hyperplastic mucosa is negative, or faintly stained for ASPH, as compared to the adjacent CRC. Red scale bar: 400 µm; yellow scale bar: 200 µm.

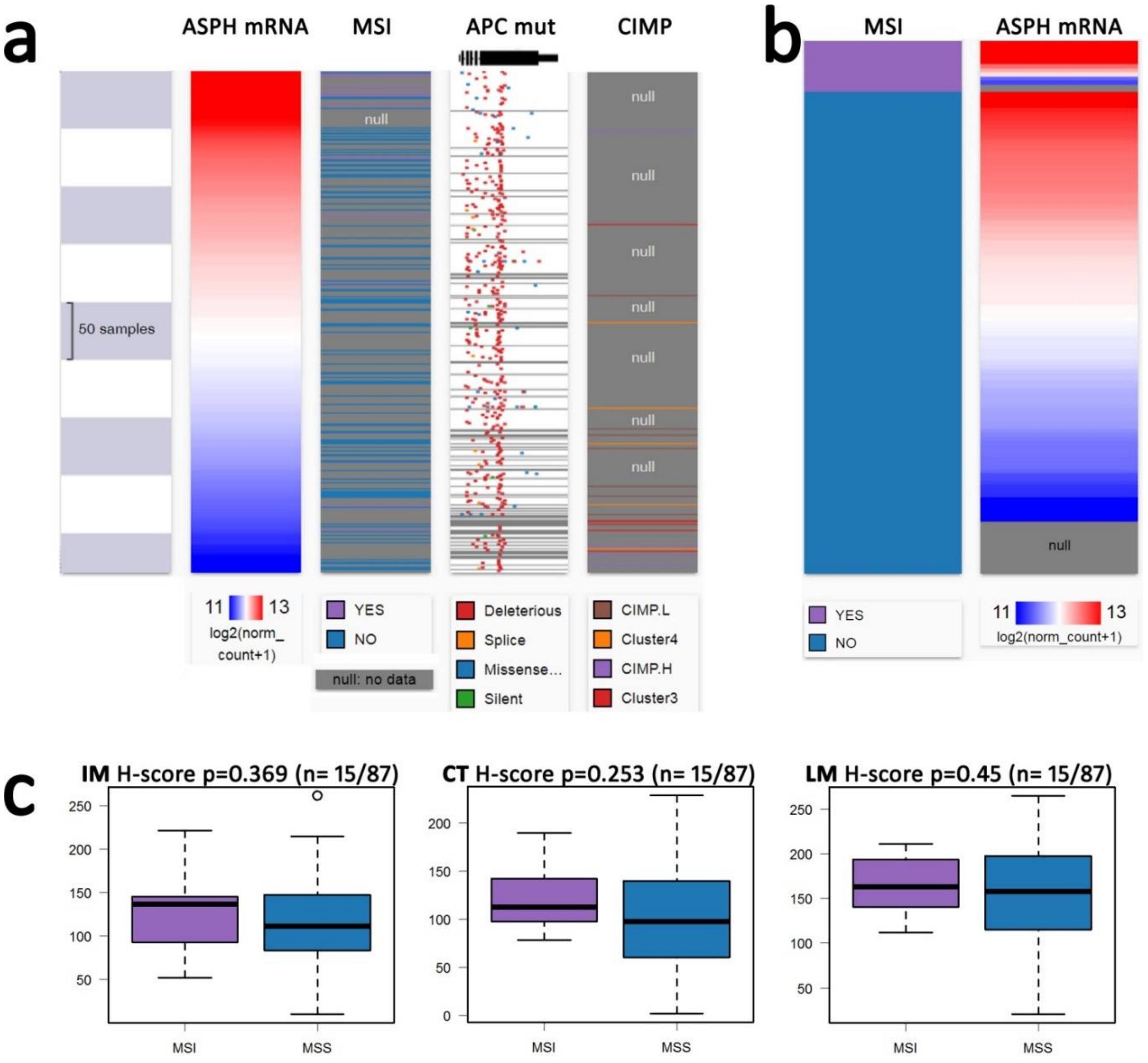

Figure S8. Analysis of ASPH levels in CRC samples with microsatellite instability (MSI): (**a**) analysis of ASPH mRNA levels in 434 TCGA-COAD CRC samples compared to MSI, APC mutation (surrogate marker for chromosomal instability, CIN) and CIMP (CpG island methylator phenotype); (**b**) A close-up of 133 samples with MSS/MSI available data (ASPH mRNA levels were available for 11 MSI samples); (**c**) Comparison of ASPH protein levels (H-Scores) in the IM, CT and LM of our samples using available data on microsatellite status (15 MSI, 72 MSS, excluded 13 not classified). The analyses shown in panels a and b were obtained using Xena functional genomics explorer (https://xenabrowser.net).

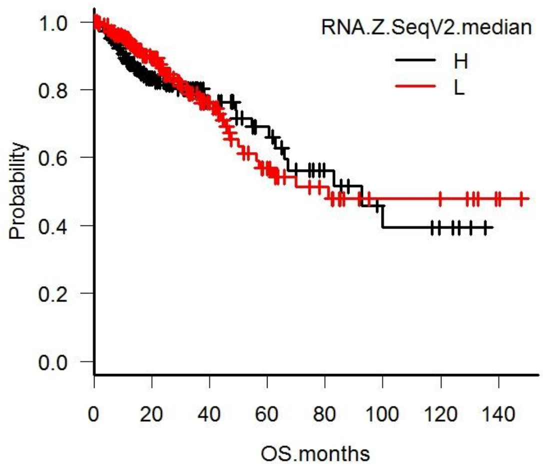

Figure S9. Kaplan-Meier analysis of overall survival in TCGA-COAD patients according to low or high ASPH mRNA levels (the median of data was used as cut-off threshold). $p = 0.735$.

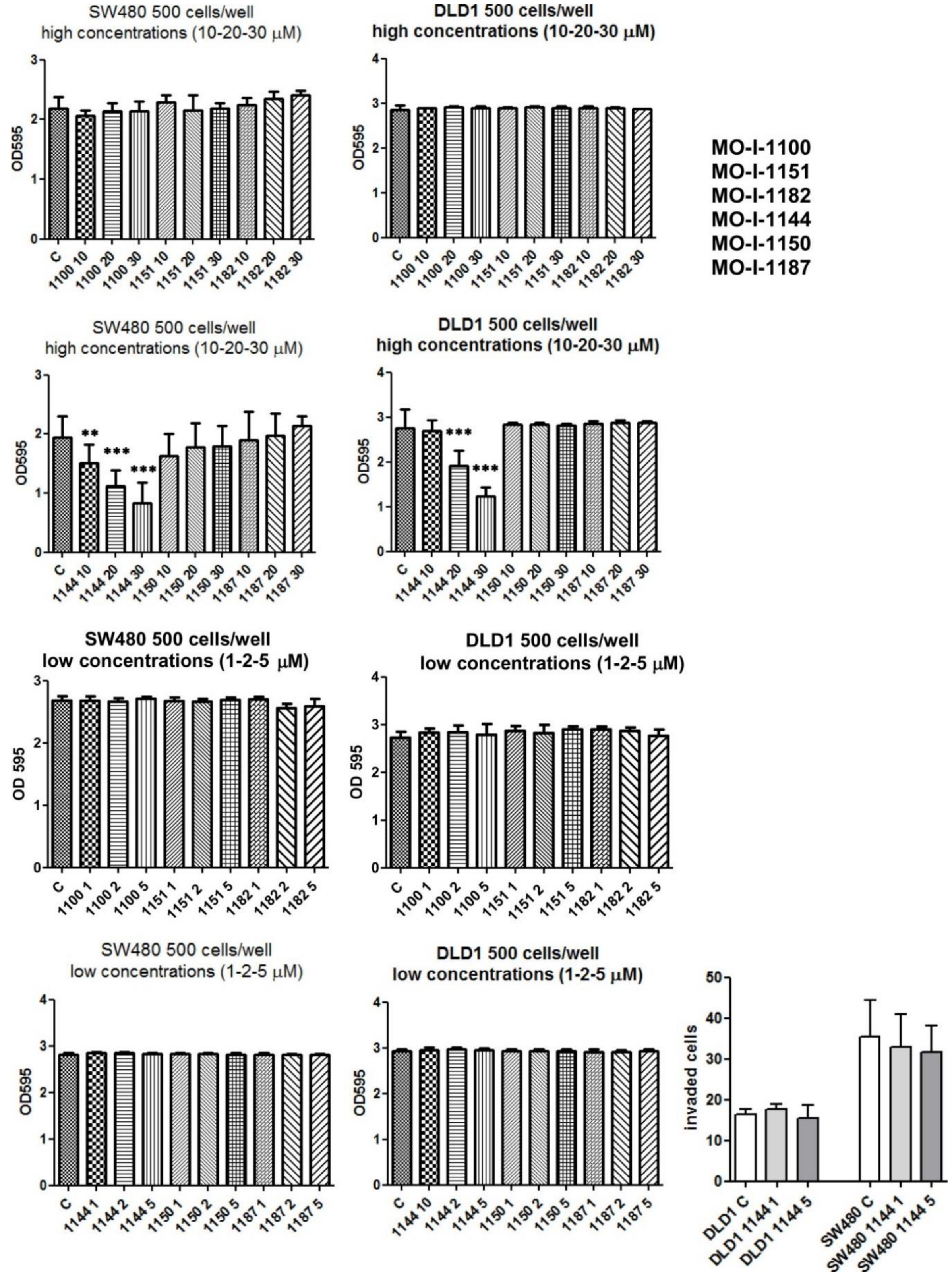

Figure S10. Screening of SMIs on ASPH catalytic activity in CRC cell lines SW480 and DLD1. A panel of ASPH inhibitors was screened to select active compounds and optimize concentrations for further analysis. Graphed data compare the effects of MO-I-1100, MO-I-1151, MO-I-1182, MO-I-1144, MO-I-1150 and MO-I-1187 at concentrations ranging from 1 to 30μM. Dose-effects of the SMIs on cell viability were measured relative to vehicle-treated control cells using the crystal violet or the invasion test. MO-I-1144 compound resulted the only effective although at high concentrations.

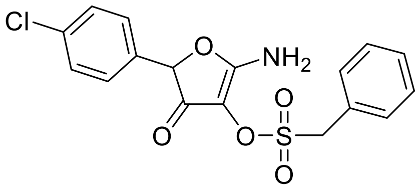

MO-I-1100

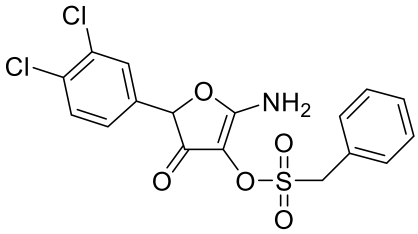

MO-I-1144

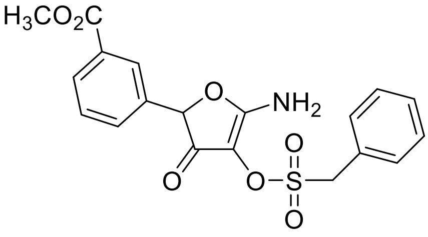

MO-I-1150

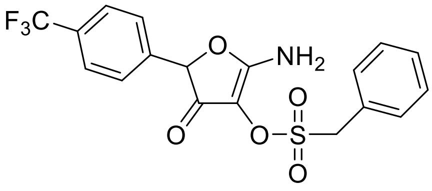

MO-I-1151

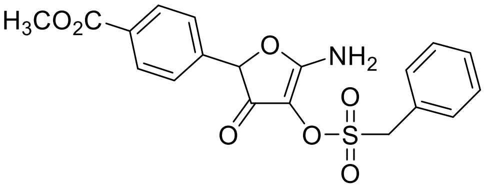

MO-I-1182

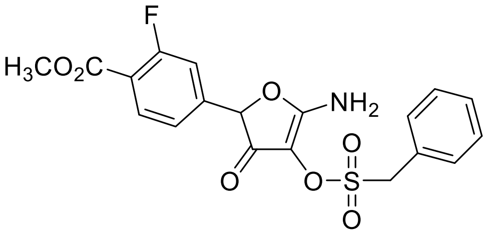

MO-I-1187

Figure S11: Structures of ASPH SMIs.

Table S1: Evaluation of Immune, NOTCH and Invasive mRNA signatures, according to ASPH levels, in COAD (Firehouse legacy) TCGA samples. Pearson's correlation r indexes with significant *p* values are color-graded according to the following scale: red r > 0.2, orange 0 < r<0.2, green -0.2 < r<0, blue r < -0.2. Significant Q values are reported in bold.

Immune signature

**ASPH** vs	**Pearson r**	**95% CI**	***p***	**Q**
CD3G	0.0509	-0.0814 0.181	0.451	0.414
CD8B	-0.108	-0.236 0.0242	0.109	0.157
CD4	-0.0321	-0.163 0.100	0.634	0.524
PTPRC-CD45	0.0802	-0.052 0.210	0.234	0.265
CD68	0.034	-0.0981 0.165	0.614	0.515
**NCR1-NKp44**	**0.178**	0.0474 0.303	**0.00786**	**0.034**
NCR2-NKp46	-0.120	-0.248 0.0115	0.0735	0.114
CTLA4	0.127	-0.00477 0.254	0.0589	0.106
**PDCD1-PD1**	**0.141**	0.00945 0.268	**0.0358**	0.083
**CD274-PDL1**	**0.307**	0.183 0.422	**3.16e-06**	**9.14e-05**

Notch signature

**ASPH** vs	**Pearson r**	**95% CI**	***p***	**Q**
ADAM10	0.123	-0.0148 0.257	0.08	0.121
ADAM17	0.075	-0.0634 0.21	0.288	0.297
**APH1A**	**0.152**	0.015 0.284	**0.03**	0.082
APH1B	-0.0962	-0.231 0.0421	0.172	0.216
ARRDC1	0.021	-0.117 0.158	0.767	0.573
CTBP1	0.0986	-0.0397 0.233	0.162	0.213
CTBP2	-0.045	-0.182 0.0933	0.524	0.466

CUL1	0.0157	-0.122 0.153	0.824	0.574
DLL1	-0.043	-0.18 0.0953	0.543	0.469
DLL3	-0.108	-0.242 0.0299	0.124	0.175
DLL4	-0.0192	-0.156 0.119	0.786	0.574
DTX1	0.0116	-0.126 0.149	0.87	0.592
DTX2	-0.0863	-0.221 0.0521	0.221	0.259
**DTX3**	**-0.169**	-0.299 -0.0316	**0.0162**	0.054
DTX3L	0.142	0.00451 0.274	0.0431	0.089
DTX4	0.00238	-0.135 0.14	0.973	0.625
EP300	-0.105	-0.239 0.0333	0.137	0.188
FBXW7	-0.0396	-0.176 0.0987	0.575	0.489
HDAC1	0.0162	-0.122 0.154	0.818	0.574
**HDAC2**	**-0.147**	-0.279 -0.00914	**0.0368**	0.083
HES1	-0.0969	-0.231 0.0414	0.169	0.216
HEYL	-0.126	-0.259 0.0117	0.0727	0.114
**ITCH**	**-0.181**	-0.311 -0.0446	**0.00966**	**0.039**
JAG1	0.0438	-0.0945 0.18	0.535	0.469
JAG2	0.0813	-0.057 0.217	0.249	0.277
**LFNG**	**-0.304**	-0.424 -0.173	**1.06e-05**	**0.0002**
MAML1	0.0298	-0.108 0.167	0.673	0.540
MAML2	0.129	-0.00844 0.262	0.0657	0.108
MAML3	0.0662	-0.0722 0.202	0.348	0.341
MFNG	0.055	-0.0834 0.191	0.436	0.406
NCOR2	0.0286	-0.11 0.166	0.686	0.543
**NCSTN**	**-0.146**	-0.278 -0.00868	**0.0374**	0.083
NOTCH1	-0.0925	-0.227 0.0458	0.19	0.230
**NOTCH2**	**0.167**	0.0302 0.298	**0.0171**	0.054
NOTCH3	-0.0677	-0.203 0.0707	0.337	0.339
NOTCH4	-0.0041	-0.142 0.134	0.954	0.620
**NUMB**	**0.240**	0.106 0.366	**0.000565**	**0.005**
NUMBL	0.122	-0.0158 0.256	0.0825	0.122
**PSEN1**	**0.214**	0.0789 0.342	**0.00214**	**0.015**
PSEN2	-0.129	-0.262 0.00844	0.0657	0.108
PSENEN	0.0237	-0.114 0.161	0.737	0.573
**RBPJ**	**0.269**	0.137 0.392	**0.000102**	**0.001**
**RBPJL**	**-0.169**	-0.3 -0.0323	**0.0158**	0.054
**RFNG**	**0.179**	0.0418 0.309	**0.0108**	**0.041**
**SNW1**	**0.330**	0.201 0.447	**1.53e-06**	**8.85e-05**
SPEN	0.130	-0.00833 0.263	0.065	0.108
HES2	-0.00536	-0.143 0.132	0.94	0.618
HES4	0.137	-0.000606 0.27	0.0511	0.098
HES7	0.0067	-0.131 0.144	0.924	0.614
HEY1	-0.012	-0.149 0.126	0.865	0.592
HEY2	-0.0798	-0.215 0.0585	0.257	0.277

Invasive signature

ASPH vs	**Pearson r**	**95% CI**	***p***	**Q**
**MMP1**	**0.190**	0.0539 0.32	**0.00656**	**0.031**
MMP2	0.0549	-0.0834 0.191	0.436	0.406
**MMP3**	**0.150**	0.0122 0.282	**0.0331**	0.083
MMP7	0.0617	-0.0766 0.198	0.382	0.368
MMP9	0.0203	-0.118 0.158	0.773	0.573
MMP10	-0.0168	-0.154 0.121	0.81	0.574
**MMP11**	**-0.229**	-0.355 -0.0939	**0.00103**	**0.008**
MMP12	0.0922	-0.0461 0.227	0.191	0.230
**MMP13**	**0.162**	0.0248 0.293	**0.0209**	0.061
**MMP14**	**0.208**	0.0719 0.336	**0.00296**	**0.015**

MMP15	-0.0486	-0.185 0.0898	0.492	0.444
MMP16	-0.000744	-0.138 0.137	0.992	0.630
MMP17	-0.0216	-0.159 0.116	0.759	0.573
MMP19	-0.0315	-0.168 0.107	0.656	0.534
MMP21	-0.0772	-0.213 0.0611	0.273	0.287
**MMP23B**	**-0.162**	-0.293 -0.0247	**0.0211**	0.061
**MMP24**	**-0.149**	-0.281 -0.0115	**0.0339**	0.083
MMP25	0.0992	-0.0391 0.234	0.159	0.213
MMP26	-0.0857	-0.221 0.0527	0.224	0.259
MMP27	0.010	-0.128 0.148	0.887	0.596
MMP28	0.134	-0.00366 0.267	0.0564	0.105
ITGB3	0.0673	-0.0711 0.203	0.34	0.339
ITGAV	0.144	0.00673 0.277	0.04	0.085
**PTK2**	**0.291**	0.16 0.413	**2.45e-05**	**0.0003**
**CDH1**	**-0.212**	-0.34 -0.0762	**0.00243**	**0.015**
CDH2	-0.0215	-0.153 0.11	0.75	0.573
SPARC	0.0174	-0.121 0.155	0.805	0.574
WFDC2	-0.0796	-0.215 0.0588	0.259	0.277
**TIMP1**	**0.138**	0.000221 0.27	**0.0497**	0.098
**TIMP2**	**0.209**	0.0732 0.337	**0.00279**	**0.015**

Table S2. ASPH silencing in combination therapy. In preliminary experiments the IC50 (half maximal inhibitory concentration) of both 5FU and Oxaliplatin, and their combination index (CI) were calculated as described (Chou TC. Theoretical basis, experimental design, and computerized simulation of synergism and antagonism in drug combination studies. Pharmacol Rev 2006; 68: 621–81). Fast forward ASPH and CTR silencing was then carried out and cells immediately plated at 3,000/well and allowed to attach. The day after, the cells were treated and the treatment lasted 96h. CI was calculated on siCTR and siASPH cells. The CI indicates a synergic effect in both ASPH silenced cell lines.

**Cell line**	**5-Fluorouracil + Oxaliplatin IC50 (μM)**	**CI**	**Effect**
SW480	8.43	0.90	
SW480 siASPH	1.77	0.47	SYNERGIC
DLD1	5.18	1.71	
DLD1 siASPH	2.93	0.57	SYNERGIC

Table S3. Patients list.

Age	Sex	Localization	UICC stage	Dukes' stage	T	N	M	G
70	F	DX	II	B2	3			2
69	F	SG	II	B2	3			2
78	M	RT	III	C2	3	1b		3
65	F	RT	I	B1	2			1
70	F	RT	II	B2	3			2
70	M	SX	II	B2	3			2
71	F	DX	I	B1	2			2
75	M	SG	I	A	1			2
54	F	SG	III	C2	3	1b		2
84	M	DX	III	C2	3	1b		3
79	M	RT	I	B1	2			2
49	F	RT	IV	D	3		1°	2
76	F	DX	III	C2	4°	1b		2
59	M	DX	IV	D	3		1°	2
84	F	DX	III	C2	3	1°		2
70	M	SG	II	B2	3			2
65	F	DX	III	C1	2	1b		3
82	F	RT	III	C2	3	2b		2
79	M	DX	III	C2	4b	2°		2
80	F	SX	II	B2	3			2

72	M	DX	II	B2	3			2
92	F	DX	III	C3	3	1°		2
69	M	SX	IV	D	4°	1b	1°	2
78	M	DX	I	B1	2			2
68	F	SG	II	B2	3			2
79	M	SG	III	C2	3	1°		2
80	M	SG	III	C2	4°	2b		3
70	F	DX	II	B2	3			2
92	M	DX	II	B2	4°			2
73	M	SX	III	C2	3	1°		2
68	F	DX	II	B2	3			2
71	F	SX	III	C2	3	1°		2
72	M	DX	II	B2	3			3
78	F	RT	I	B1	2			1
58	F	SX	III	C2	3	1°		1
78	F	RT	II	B3	4b			2
74	M	RT	IV	D	3		1°	2
63	F	RT	I	B1	2			2
54	M	DX	III	C2	3	1b		3
80	F	SG	III	C2	4°	2°		2
88	M	DX	III	C2	3	1°		2
64	F	RT	II	B2	3			2
42	M	SG	IV	D	4°	2°	1°	2
75	M	DX	IV	D	3	2b	1b	2
75	F	SG	II	B2	3			3
68	F	DX	II	B2	3			2
83	M	DX	I	B1	2			2
59	M	SX	III	C2	3	1°		2
84	F	DX	III	C2	4°	1b		2
74	M	SG	III	C2	3	2°		2
65	F	SG	I	B1	2			2
68	M	DX	III	C2	3	2°		2
52	F	RT	IV	D	4°	2°	1°	2
59	F	DX	II	B2	4°			2
66	M	DX	IV	D	4°	1b	1°	2
76	M	SG	I	B1	2			2
72	M	SX	II	B2	3			1
55	M	SG	III	C2	3	1°		3
81	M	SX	III	C2	3	1°		2
72	M	SX	III	C2	3	1°		2
60	M	RT	I	B1	2			2
85	F	DX	II	B2	3			2
80	F	DX	I	B1	2			3
78	F	DX	I	B1	2			2
42	F	RT	III	C1	2	1b		2
71	F	DX	II	B2	3			2
84	F	DX	II	B2	3			2
46	F	SX	III	C2	4b	2°		3
65	M	SX	IV	D	4°	1b	1°	2
63	M	RT	III	C2	3	1b		2
59	M	RT	II	B2	3			2
73	F	DX	II	B2	3			2
52	M	SG	II	B2	3			3
81	F	RT	III	C2	3	1b		2
64	M	SX	IV	D	3		1°	2
85	M	RT	II	B2	3			1
59	F	DX	III	C1	2	1°		2
90	M	DX	III	C2	3	2b		2
54	F	TR	II	B2	3			2

81	M	DX	II	B2	4°			2
59	M	SX	I	B1	2			2
68	M	RT	II	B2	4b			2
78	M	SG	II	B2	4°			2
75	F	DX	II	B2	3			1
41	F	DX	III	C2	3	2b		3
73	M	DX	III	C2	3	2b		3
82	F	SX	III	C2	3	1b		2
57	F	SX	III	C2	3	1°		2
80	M	SG	IV	D	4°	2b	1°	3
75	M	SG	II	B2	3			2
82	F	DX	III	C2	3	2b		3
61	F	TR	III	C2	3	1°		2
67	F	SG	IV	D	3	1b	1°	3
54	F	SX	II	B2	3			3
63	M	DX	III	C2	3	1°		3
79	M	DX	III	C2	4°	2b		3
86	F	DX	II	B2	3			2
55	M	RT	IV	D	4°	2b	1°	2
69	F	DX	III	C2	3	1°		2
92	F	RT	II	B2	3			2

© 2020 by the authors. Licensee MDPI, Basel, Switzerland. This article is an open access article distributed under the terms and conditions of the Creative Commons Attribution (CC BY) license (http://creativecommons.org/licenses/by/4.0/).
